# Singleplex, multiplex and pooled sample real-time RT-PCR assays for detection of SARS-CoV-2 in an occupational medicine setting

**DOI:** 10.1038/s41598-022-22106-2

**Published:** 2022-10-22

**Authors:** Kimberly S. Butler, Bryan D. Carson, Joshua D. Podlevsky, Cathryn M. Mayes, Jessica M. Rowland, DeAnna Campbell, J. Bryce Ricken, George Wudiri, The SNL COVID Dx Assay Team, Jesse Cahill, Dulce Hayes, Tessily N. Hogancamp, Danae M. Maes, Daniella V. Martinez, Amanda S. Peretti, Stephenie A. Roberson, Anne M. Ruffing, Leslie M. Huggins, Taylor Settecerri, Chuck R. Smallwood, Matthew S. Tezak, Linda Winona, Dongmei Ye, Jerilyn A. Timlin

**Affiliations:** 1 Molecular and Microbiology Department, Sandia National Laboratories, Albuquerque, NM 87123 USA; 2 WMD Threats and Aerosol Science, Sandia National Laboratories, Albuquerque, NM 87123 USA; 3 Global Chemical and Biological Security, Sandia National Laboratories, Albuquerque, NM 87123 USA; 4 Biological and Chemical Sensors Department, Sandia National Laboratories, Albuquerque, NM 87123 USA; 5 Cooperative Nuclear Counterproliferation, Sandia National Laboratories, Albuquerque, NM 87123 USA; 6 Computational Biology and Biophysics Department, Sandia National Laboratories, Albuquerque, NM 87123 USA; 7 Electronic, Optical, and Nano Department, Sandia National Laboratories, Albuquerque, NM 87123 USA; 8 System and Component Reliability, Sandia National Laboratories, Albuquerque, NM 87123 USA

**Keywords:** Viral infection, Diseases, Laboratory techniques and procedures, Occupational health

## Abstract

For workplaces which cannot operate as telework or remotely, there is a critical need for routine occupational SARS-CoV-2 diagnostic testing. Although diagnostic tests including the CDC 2019-Novel Coronavirus (2019-nCoV) Real-Time RT-PCR Diagnostic Panel (CDC Diagnostic Panel) (EUA200001) were made available early in the pandemic, resource scarcity and high demand for reagents and equipment necessitated priority of symptomatic patients. There is a clearly defined need for flexible testing methodologies and strategies with rapid turnaround of results for (1) symptomatic, (2) asymptomatic with high-risk exposures and (3) asymptomatic populations without preexisting conditions for routine screening to address the needs of an on-site work force. We developed a distinct SARS-CoV-2 diagnostic assay based on the original CDC Diagnostic Panel (EUA200001), yet, with minimum overlap for currently employed reagents to eliminate direct competition for limited resources. As the pandemic progressed with testing loads increasing, we modified the assay to include 5-sample pooling and amplicon target multiplexing. Analytical sensitivity of the pooled and multiplexed assays was rigorously tested with contrived positive samples in realistic patient backgrounds. Assay performance was determined with clinical samples previously assessed with an FDA authorized assay. Throughout the pandemic we successfully tested symptomatic, known contact and travelers within our occupational population with a ~ 24–48-h turnaround time to limit the spread of COVID-19 in the workplace. Our singleplex assay had a detection limit of 31.25 copies per reaction. The three-color multiplexed assay maintained similar sensitivity to the singleplex assay, while tripling the throughput. The pooling assay further increased the throughput to five-fold the singleplex assay, albeit with a subtle loss of sensitivity. We subsequently developed a hybrid ‘multiplex-pooled’ strategy to testing to address the need for both rapid analysis of samples from personnel at high risk of COVID infection and routine screening. Herein, our SARS-CoV-2 assays specifically address the needs of occupational healthcare for both rapid analysis of personnel at high-risk of infection and routine screening that is essential for controlling COVID-19 disease transmission. In addition to SARS-CoV-2 and COVID-19, this work demonstrates successful flexible assays developments and deployments with implications for emerging highly transmissible diseases and future pandemics.

## Introduction

In 2020, the outbreak of the coronavirus disease 2019 (COVID-19) caused by the novel severe acute respiratory syndrome coronavirus 2 (SARS-CoV-2) resulted in a sudden and rapid increase in hospitalizations for pneumonia. The worldwide spread of SARS-CoV-2 was extremely swift. The outbreak officially began on December 31, 2019 when China alerted the WHO of a cluster of pneumonia cases with unknown origin, later determined by the WHO on January 7, 2020 to be caused by a novel coronavirus^1^. The earliest case outside of China was confirmed January 13, 2020 in Thailand, and the first US case was confirmed January 20, 2020^1–3^. Human-to-human transmission was confirmed, and on January 30, 2020 the WHO declared COVID-19 a Public Health Emergency of International Concern^2^. The US Centers for Disease Control and Prevention (CDC) stated on February 25, 2020 that COVID-19 met 2 of the 3 factors for classification as a pandemic, specifically illness resulting in death and sustained person-to-person spread. The only criteria missing at that time was worldwide spread. However, COVID-19 continued to progress and on March 11th the WHO declared COVID-19 a pandemic^1^. By May 9, 2020, more than 4 million COVID-19 cases were reported globally, and the case count rapidly increased in less than two months to more than 10 million people by June 29, 2020^1^.

In the USA, the president declared COVID-19 a National Emergency on March 13, 2020, and that same month, US states and territories began issuing various community mitigation policies to reduce the transmission of COVID-19^4^. One of the most widely implemented strategies were stay-at-home orders, with 42 states and territories issuing some form of order affecting 73% of US counties between March 15 and May 31, 2020^4^. The use of contact and travel restrictions, social distancing measures, as well as school and work closures in the USA was supported by the prior success in China to impede COVID-19 disease transmission^5,6^. Epidemiological simulations and models suggested that these community mitigation policies might be required for months or repeatedly enacted^7–10^. Studies additionally highlighted the need for early detection to control rampant COVID-19 transmission^2,5,8^.

For workplaces which cannot operate solely in a telework or remote work capacity, there was a critical need for occupational SARS-CoV-2 diagnostic testing^11–13^. Non-telework compatible workplaces included the obvious environments of medical providers as well as the less obvious environments of critical infrastructure and utilities, national laboratories, security, fire, and rescue. In the USA, a SARS-CoV-2 nucleic acid diagnostic test created by the CDC (CDC 2019-Novel Coronavirus (2019-nCoV) Real-Time RT-PCR Diagnostic Panel (EUA200001)) was permitted Emergency Use Authorization (EUA) by the Food and Drug Administration (FDA) on February 4, 2020—a month prior to the pandemic declaration by the WHO^14,15^. While the CDC Diagnostic Panel was authorized for use, it exclusively specified a small set of highly specific reagents and equipment, generating extremely high demand and limited availability for occupational diagnostics^16^. Furthermore, occupational settings necessitate rapid test results—ideally less than 2 days—for effective contact tracing, mitigating workplace transmission, and maintaining essential workplace capabilities^10,13,17^. Early in the pandemic and continuing into the summer of 2020, the time from patient sample collection to real time RT-PCR testing to result reporting was 4–7 days^18,19^. The shortage of available diagnostic testing reagents and equipment during the ongoing pandemic necessitated sample prioritization; the highest priority reserved for hospitalized patients and healthcare workers with known exposures. Testing priority and limitation to a select subset of the highest risk individuals increased the risk of occupational transmission^16^.

To address the needs of essential workers, we developed an onsite occupational testing facility capable of near 24-h turnaround time from sample collection to result reporting. Our occupational testing facility established a distinct SARS-CoV-2 nucleic acid diagnostic test EUA to avoid ongoing supply chain issues as well as reagent and equipment scarcity. To maintain adequate testing throughput, we developed a multiplexed assay and pooling strategies to compensate for continuously increasing testing loads. In this manuscript, we detail the development of our SARS-CoV-2 nucleic acid diagnostic assays and provide use cases for singleplex, multiplex, and pooled sample assessment in an occupational setting during the COVID-19 pandemic. These methods serve as a model for future diagnostic testing needs in occupational settings.

## Methods

### Clinical samples

For initial assay development, known positive and negative nasopharyngeal samples were obtained from external clinical laboratories; all samples obtained from other clinical laboratories were anonymized prior to being received and no patient information was provided to our laboratory.

After the initial assay development, clinical nasopharyngeal samples from our workforce were obtained for clinical analysis from on-site medical providers and anonymized upon receipt to protect patient privacy throughout the testing process. Due to the occupational nature of the testing, samples were collected from symptomatic individuals, asymptomatic individuals with known contact with a COVID positive person and individuals with travel requiring testing prior to return to work. Samples were collected from April 2020 through June 2021 for on-site occupational medicine analysis. After analysis, clinical results were linked with patient data and were transmitted to the medical providers. After clinical processing, the remaining sample was stored at − 80 °C for use in assay development. Aggregated patient data without personal identifiable information was also retained for data analysis.

Clinical samples were assessed using the SNL-NM 2019 nCoV Real-Time RT PCR Diagnostic Test EUA Summary (EUA200481), and details for this assay are available within this EUA^20^. All methods were performed in accordance with the relevant guidelines and regulations. Methods are also summarized below for clarity.

### RNA extraction

RNA extraction from nasopharyngeal samples was performed using the Quick-RNA Viral Kit (Zymo Research) combined with a vacuum manifold (Qiagen) and described briefly below. A human virus negative extraction control (NEC) made of A549 cells (ATCC) was utilized in all extractions as a control for extraction quality. 140 µL of nasopharyngeal swab matrix or NEC was combined with 400 µL Viral RNA buffer and placed in a Zymo-Spin IC column and loaded onto the vacuum manifold using disposable connectors to reduce contamination risk. Under vacuum, columns were washed twice with 500 µL Viral RNA Wash Buffer and once with 750 µL 100% ethanol. Columns were removed from vacuum and then centrifuged (10,000 rcf, 2 min) to remove all residual ethanol. RNA was then eluted in 20 µL of nuclease free water (ThermoFisher) utilizing centrifugation (10,000 rcf, 2 min). RNA samples were kept on ice and utilized immediately for PCR.

### Singleplex RT-PCR

One step RT-PCR was performed using the previously developed qPCR primers and probes which includes two primer/probe sets targeting the SARS-CoV-2 nucleocapsid protein gene (N1 and N2) and a primer/probe set targeted to human RNase P mRNA (RP) to serve as an internal sample control (2019-nCoV CDC qPCR Probe Assay CDC Emergency Use Authorization Kits, Integrated DNA Technologies, Inc.)^14^ and TaqMan Fast Virus 1-step Master Mix (Ambion, ThermoFisher). A SARS-CoV-2 standard (COV019, Exact Diagnostics) was utilized as a positive control, and nuclease free water was utilized for no template control (NTC). All RT-PCR reactions consisted of 1.5 µL combined primer/probe mix for a given target (N1, N2, or RP), 5 µL TaqMan Fast Virus 1-step Master Mix, 8.5 µL nuclease free water and 5 µL RNA. Reactions were prepared in 96 well PCR optical plates (Applied Biosystems). Thermocycling conditions were: 25 °C 2 min; 50 °C 15 min; 95 °C 2 min followed by 45 cycles of 95 °C 15 s, 55 °C 60 s. Baseline was set to 1–8 cycles. Initial assay development was performed on an ABI 7500 instrument (Applied Biosystems, Inc.), but was bridged to 3 additional machines: Quant Studio 5 (Applied Biosystems, Inc.), CFX96 touch (Bio-Rad), and CFX Connect (Bio-Rad) to increase testing capacity by increasing the types of machines available. Bridging data comparing the instruments is provided within the SNL-NM 2019 nCoV Real-Time RT PCR Diagnostic Test EUA Summary (EUA200481)^20^.

### Interpretation of clinical samples

All clinical samples were interpreted as per the SNL-NM 2019 nCoV Real-Time RT PCR Diagnostic Test EUA Summary (EUA200481)^20^. Briefly, clinical samples can only be interpreted in a valid assay with valid controls. Positive control must demonstrate a Ct ≤ 38 for RP, N1 and N2. The NEC must demonstrate no detectable signal in N1 or N2 and RP with a Ct ≤ 38. The NTC must demonstrate no detectable signal in N1, N2 or RP. Once the assay has been determined to be valid, clinical specimens can be interpreted as per Table 1.

**Table 1:** Clinical specimen interpretation.

SARS-CoV-2 N1	SARS-CoV-2 N2	RP	Results interpretation
+	+	+/–	SARS-CoV-2 positive
If only one of the two targets are positive		+/–	Inconclusive
–	–	+	SARS-CoV-2 not detected
–	–	–	Invalid

The first time a sample is invalid or inconclusive, the sample is rerun, the second time the sample is recorded and reported as inconclusive or invalid. For any sample reported as inconclusive or invalid a new sample was requested for analysis.

### Singleplex limit of detection (LoD) studies

A preliminary LoD was determined by testing twofold dilutions (250 copies/reaction–7.8 copies/reaction) of synthetic RNA (SARS-CoV-2 standard, COV019, Exact Diagnostics) spiked into pooled, negative nasopharyngeal swab samples using 3 replicates at each target level. Spiked levels were calculated based on the final extraction volume of 20 µL and assumed perfect recovery through the extraction process. Samples were extracted and PCR was performed using the protocol described above.

Following initial LoD determination, a more comprehensive LoD study was performed to define the LoD of the assay. A set of 20 samples spiked at 15.6 copies/reaction and 40 individual samples spiked at 31.3 copies/reaction were created using 140 µL of a pooled, negative nasopharyngeal swab background. Samples were extracted and PCR performed using the protocol above.

Once the LoD was defined, a blinded study covering a range of LoD was performed. 30 individual non-pooled, negative and 30 individual non-pooled, spiked positive samples were created. Spikes were prepared for each sample containing either nuclease-free water (negative samples) or synthetic RNA (SARS-CoV-2 standard, COV019, Exact Diagnostics, positive samples). The volume of the spike addition was kept constant at 40 µL for all samples. The samples were randomized and included 1 × LoD (10 samples), 2 × LoD (10 samples), 4 × LoD (5 samples) and 8 × LoD (5 samples) along with the negative samples. Samples were assessed using the standard protocol for RNA extraction and singleplex PCR.

### Multiplex assay development

To allow simultaneous detection of 3 targets in a single well, the fluorophores on two of the probes, N2 and RP, were changed from the original SNL-NM 2019 nCoV Real-Time RT PCR Diagnostic Assay (EUA200481)^20^, which utilized only a single fluorophore (FAM) to SUN and ATTO 647 respectively. The sequences were not altered and the N1 probe was maintained as FAM labeled. As the use of multiplexed probes requires the creation of a custom primer and probe mix, the primers and probes were bought individually (Integrated DNA Technologies, Inc.). The concentrations of the N1 and N2 primers and probes were maintained from the original assay (500 nM and 125 nM for primers and probes respectively). To determine the appropriate concentration of the RP primers and probe within the multiplex format, the concentrations of primers and probe were systematically varied and tested within the assay. The concentration of the RP primers was varied from 500 to 125 nM and the probe concentration was varied from 125 to 31.25 nM. Optimal concentration of RP primers and probe for the multiplexed assay was determined to be 166 nM and 41.7 nM for primers and probe, respectively.

Sample processing (PCR settings, cycle conditions and detection), results interpretation (controls and Ct cut off for positive samples), and LoD were all maintained the same from the singleplex assay to the multiplex sample strategy.

### Pooled assay assessment

To create pooled samples with known statuses, 25 samples previously determined to be positive and 110 samples previously determined to be negative by the SNL-NM 2019 nCoV Real-Time RT PCR Diagnostic Assay (EUA200481) were used to create 5-sample pools. Samples were pooled prior to processing. Positive samples were selected to cover the signal range seen within the occupational population and ranged from Ct < 20 through ~ 37. In addition to those expected to reflect the positive population, 7 samples of high Ct (34–38) were selected to examine the effect of pooling on weak positive samples. Expected positive 5-sample pools were created by combining 100 µL of one individual positive patient sample with 100 µL from each of 4 unique negative patient samples. This was done for all positive patient samples thereby creating 25 5-sample pools (i.e., a total of 25 positive samples combined with 100 negative samples). Expected negative 5-sample pools were created by combining 100 µL from each of 5 unique negative patient samples utilizing 110 negative samples to create 22 expected negative 5-sample pools.

Testing of all 5-sample pools was done following the standard procedure for the SNL-NM 2019 nCoV Real-Time RT PCR Diagnostic Assay (EUA200481). The assay was performed blinded by a technician who was provided no knowledge of the pool composition or any prior analysis. For test development, inconclusive samples were not reanalyzed. However, for use in the clinical setting, all positive and inconclusive pools would have each sample present in the pool rerun individually utilizing the singleplex assay.

The PCR settings including  the number of cycles and detection criteria were maintained from the singleplex assay to the pooled sample strategy. Interpretation of the results was also kept the same as the singleplex assay, including the controls and the cut off for determining a positive sample. The LoD of the 5-sample pooled assay was determined using the same procedures as the singleplex assay.

#### Typical workflow in occupation use setting

The standard timeline in our occupational setting was as follows. Personnel with symptoms, known contact with COVID positive individuals or travelers requiring testing prior to return to work would call the onsite occupational medical facility and would be provided an appointment for sample collection the same day or the following day. Samples would be collected throughout the workday, typically 9–3 pm and then transported by courier to the diagnostic laboratory. A sample intake team would formally intake the samples, assign them unique sample identification numbers and set up the sample run order based on priority from 4 to 7 pm. The next day, one or more diagnostic assay teams would perform the assay starting at 8 am, depending on the number of samples and their priority ranking. Symptomatic personnel were highest priority for testing, followed by those with known contact, and those needing testing due to travel were the lowest priority. The diagnostic assay teams would provide the results to a reporting team and the reporting team would confirm the assay results, generate formal reports for each patient, and provide those to the occupational medicine providers, with the last reports sent prior to 3 pm. The occupational medicine providers would provide reports to the patients and initiate contact tracing prior to end of the workday. On average, the time from sample collection to report was ~ 1.5 days and the time from samples arriving to the diagnostic lab to report was < 1 day.

### Ethics approval and consent to participate

All testing was done as part of clinical laboratory operations and is presented only in aggregate and anonymized form within this manuscript. The use of the retrospective data in this study is exempt from ethics approval and consent to participate. The decision for this exemption was made by the Sandia National Laboratories Human Studies Board.

## Results

### Singleplex assay analytical sensitivity and clinical validation

**Figure 1:**
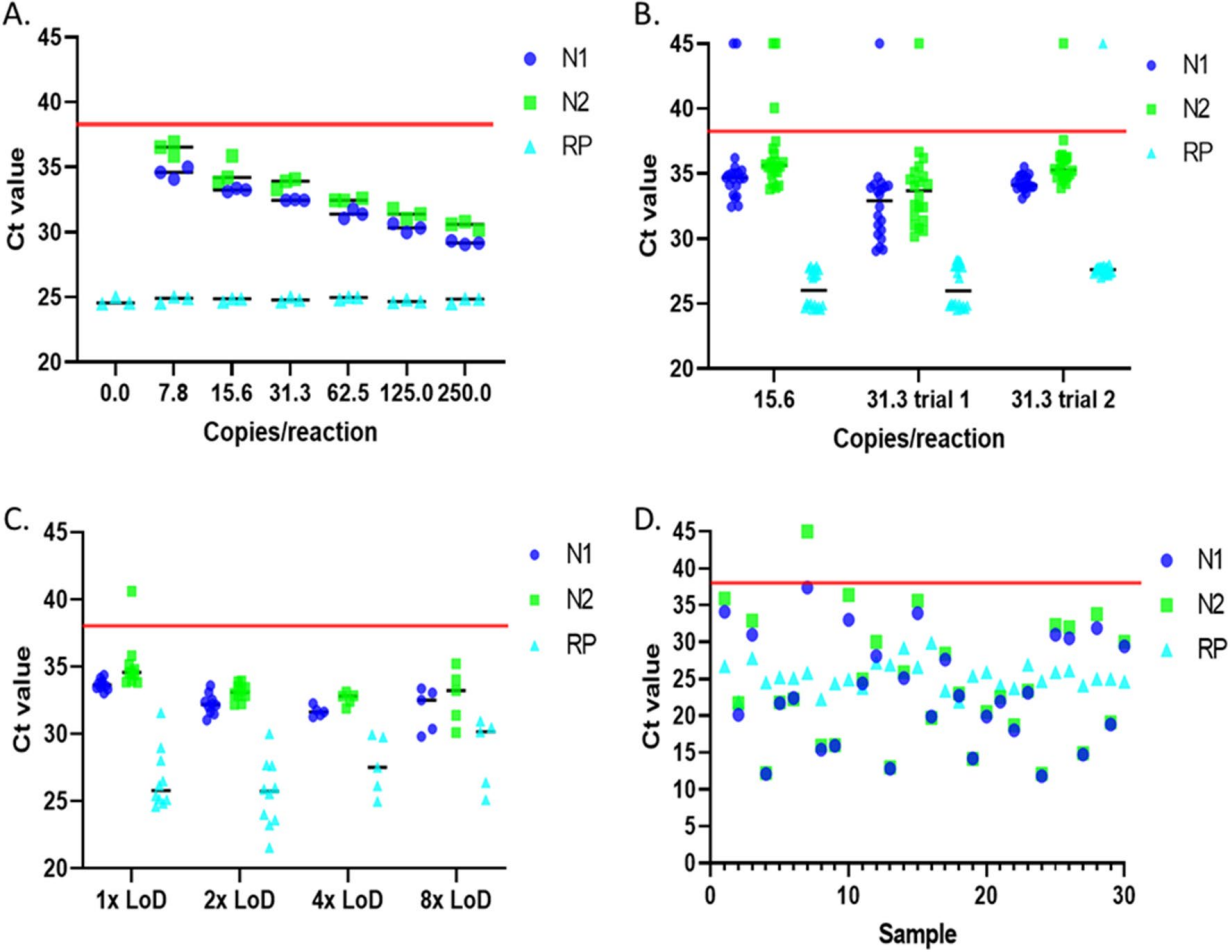
SARS-CoV-2 RT-PCR assay development. Assay detects 2 sequences (N1 and N2) from SARS-CoV-2 and a human RNA (RP) as an internal extraction control. (**A**) Rough Limit of detection (LoD) of positive control RNA spiked into pooled negative nasopharyngeal samples at known copy numbers. Black lines represent the mean, N = 3. The red line denotes the Ct cutoff (38) for a positive result. (**B**) Comprehensive LoD study of positive control RNA spiked into pooled negative nasopharyngeal samples at known copy numbers. These studies defined 31.25 copies/reaction as the LoD. Black lines represent the mean, N = 20. The red line denotes the Ct cutoff (38) for a positive result. (**C**) Mock clinical samples created by spiking known concentrations of synthetic SARS-CoV-2 RNA into individual negative nasopharyngeal samples. Samples were blinded to assay personnel. N = 10 for 1 × and 2 × LoD. N = 5 for the 4 × and 8 × LoD. The red line denotes the Ct cutoff (38) for a positive result. (**D**) 30 positive clinical confirmation samples. Samples were blinded to assay personnel and run concurrently with negative samples. The red line denotes the Ct cutoff (38) for a positive result. Samples with no signal detected during the real time RT-PCR are shown at Ct 45 in the graphs.

The initial testing focused on determining the analytical sensitivity and the results of an initial Limit of Detection (LoD) study using pooled negative nasopharyngeal swab samples spiked with known amounts of synthetic SARS-CoV-2 RNA are shown in Fig. 1A. For all samples, human extraction control (RP) exhibited stable Ct values regardless of the amount of SARS-CoV-2 synthetic RNA spiked. As the number of copies of SARS-CoV-2 RNA decreased, the N1 and N2 Ct values increased, but all spiked samples were successfully detected (Ct ≤ 38 for both N1 and N2). In all cases, the Ct value for N2 was slightly higher than the Ct value for N1, even though identical amounts of the target were present. As both the 15.6 and 31.25 copies/reaction averages in the initial LoD were greater than 2 Ct less than the Ct = 38 cutoff for both N1 and N2 (N1 = 33.2 and N2 = 34.6, N1 = 32.5 and N2 = 33.8 for 15.6 and 31.3 copies respectively), 15.6 and 31.3 copies/reaction were chosen for a more comprehensive study to identify the LoD.

The results of the comprehensive study which examined 10 spikes each at both 15.6 and 31.25 copies/reaction performed in duplicate for a total of 20 samples per spike amount are shown in Fig. 1B. Examining the 15.6 copies/reaction data, 17 samples were successfully detected as positive (Ct < 38 for both N1 and N2), 2 samples were undetected, and one sample was inconclusive (Ct N1 < 38 and N2 > 38). In comparison, the 31.25 copies/reaction data showed successful detection in 19 of 20 samples with 1 sample undetected. To confirm an LoD of 31.25 copies/reaction, a confirmation study was done with an additional 20 contrived samples (31.25 trial 2, Fig. 1B). In the second study, 19 of 20 samples were again successfully detected as positive and one sample was inconclusive (N1 < 38, N2 > 38 and RP > 38).

Following identification of the LoD, a series of 30 positive and 30 negative blinded samples covering a range of LoDs (1 × to 8 × LoD) and negative samples designed to explore the range of response in mock clinical samples were assessed. All 30 negative samples were correctly identified as negative, resulting in a negative percent agreement of 100% (95% confidence interval 88.43%—100%). At concentrations of 2x, 4x and 8x the LoD, 100% of the samples were correctly identified as positive (Fig. 1C). At the 1 × LoD, one sample was inconclusive (N1: 33.7, N2: 40.6, RP: 24.8). Therefore, 29 of 30 samples were correctly identified as positive, resulting in a positive percent agreement of 96.67% (95% confidence interval 82.78–99.92%).

Finally, 30 positive and 30 negative clinical samples from other laboratories that had been previously assessed using an already validated test were run to provide validation in a true clinical setting. All 30 negative samples were correctly identified as negative, resulting in a negative percent agreement of 100% (95% confidence interval 88.43–100%). The positive samples ranged from strongly positive with Ct values between 10 and 15 to weak positives with Ct greater than 32 (Fig. 1D). Of the positive samples, 29 were correctly identified as positive and one sample was identified as inconclusive (N1: 37.4, N2: not detected, RP: 25.8). The inconclusive sample was the weakest positive examined. Therefore, 29 of 30 samples were correctly identified as positive, resulting in a positive percent agreement of 96.67% (95% confidence interval 82.78–99.92%).

### Positivity trends

**Figure 2:**
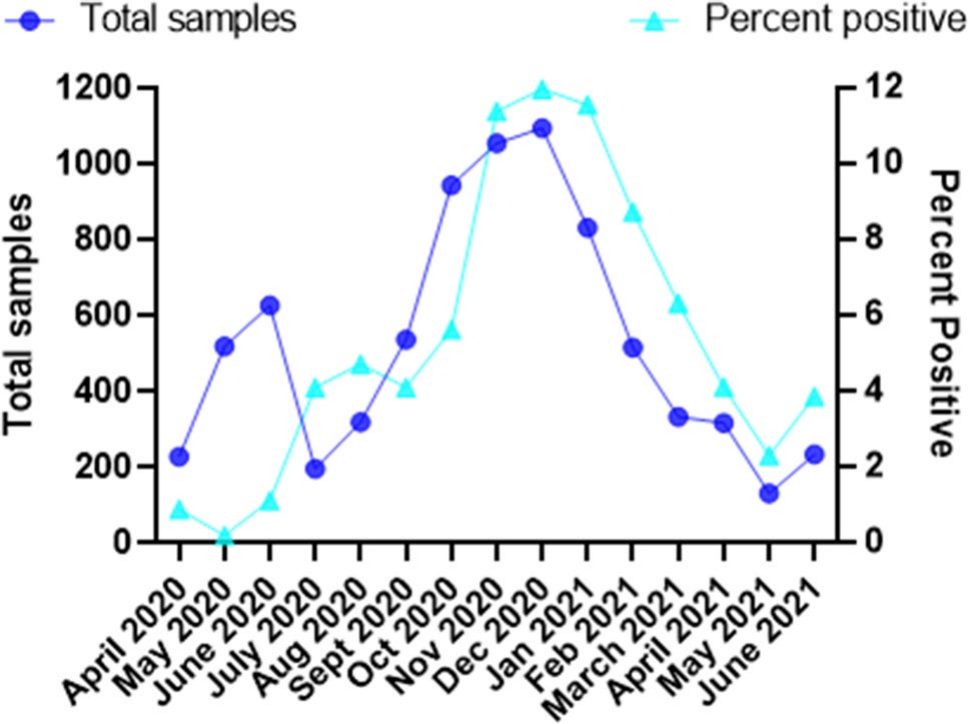
SARS-CoV-2 RT-PCR Occupational testing using the Sandia National Laboratories SNL-NM 2019 nCoV Real-Time RT PCR Diagnostic Assay (EUA200481). Total occupational samples tested between April 2020 and June 2021 and the percent positive of these samples.

During the initial months of the COVID-19 pandemic, both the testing load and the positivity rate was low (Fig. 2). Starting in July 2020, the positivity rate increased, and the needed testing volume began to increase the following month. The positivity rate peaked in November 2020 at ~ 11 to 12% and continued at this rate through January 2021. The daily sample volume peaked in November and December of 2020. This trend confirmed the need for a high throughput assay allowing for faster response time to increases in COVID 19 prevalence in the occupational population.

### Multiplex assay development, analytical sensitivity and clinical validation

A multiplex assay format was developed that would allow 93 samples to run in a single 96-well PCR plate by detection of all 3 targets in a single well while minimizing changes to the existing singleplex assay. Initially, the assay was tested with the primers and probes at the same concentration as the original EUA with positive control spiked into pooled negative nasopharyngeal samples at 1 to 4 × LoD (Supplemental Fig. 1a). In the singleplex format, the N1 and N2 signals showed a slight delay of approximately 1 Ct between N1 and N2 detection (Fig. 1A). In contrast, the N2 signal was significantly delayed (~ 4–5 Ct) in the multiplex assay. No delay was noted in the positive control, which has the equivalent amount of SARS-CoV-2 synthetic RNA as the 4 × LoD but much lower levels of RP than clinical samples. To test the hypothesis that limiting the RP primers and probes would result in increased performance, experiments were conducted with the RP primers and probe reduced to 1/2, 1/3 and 1/4 the amount of the N1 and N2 primers and probes and the Ct of N1 and N2 in contrived samples spiked at 2 × LoD was assessed (Supplemental Fig. 1b). Reduction of the RP primers and probe to 1/3 the level of the N1 and N2 primers and probes resulted in the closest fit to the ideal line and was selected for further testing.

**Figure 3:**
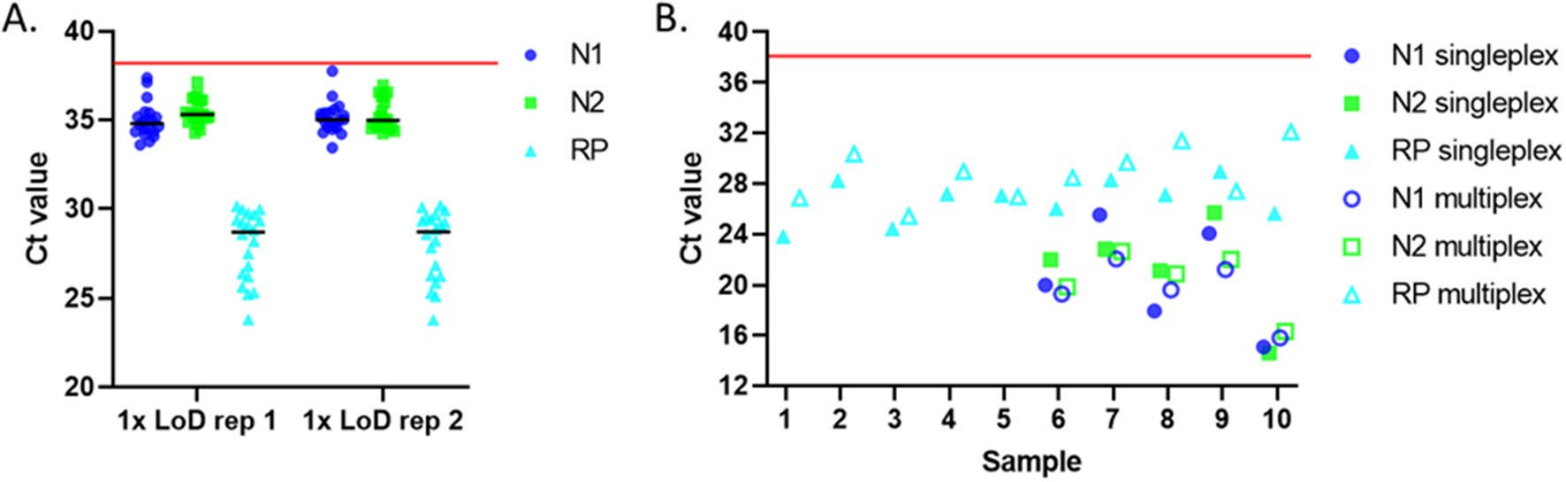
SARS-CoV-2 RT-PCR multiplex assay development. Assay detects 2 sequences (N1 and N2) from SARS-CoV-2 and a human RNA (RP) as an internal extraction control. (**A**) Mock clinical samples created by spiking SARS-CoV-2 synthetic RNA at the level of 1 × LoD into individual negative nasopharyngeal samples. N = 20 per technical replicate. The black lines denote the means for each target and replicate. The red line denotes the Ct cutoff (38) for a positive result. (**B**) 5 negative and 5 positive clinical samples were assessed and compared to the singleplex detection. Samples were blinded to assay personnel. The red line denotes the Ct cutoff (38) for a positive result.

The initial LoD test was repeated with RP primers and probes at 1/3 the concentration as the original assay (Supplemental Fig. 1c). With the concentration of the RP primers and probe more limited, all levels of spiked synthetic SARS-CoV-2 RNA were detected as positive, including the 1 × LoD. Interestingly, the N2, which was delayed in the singleplex assay compared to N1 now appears with a nearly identical Ct. Next, 20 individual negative nasopharyngeal spiked with synthetic SARS-CoV-2 RNA at the previously defined 1 × LoD were tested. As the extraction methodology was not changed and yielded 20µL, each sample was run twice to create technical replicates (Fig. 3A). In both technical replicates of 20, all 20 samples were correctly identified as positive.

Finally, a series of clinical samples which had previously been assessed via the singleplex assay were tested in the multiplex assay format. Five unique positive and 5 unique negative samples were assessed blinded. All 10 samples were correctly identified as positive or negative using the multiplex assay and both the positive and negative agreement was 100% (95% confidence interval 78.3–100%). The Ct values of the singleplex assessment were compared to the multiplex assay to determine if multiplexing had any effect on the detection (Fig. 3B). No pattern was observed in the detection of N1 and N2. However, 80% of the samples showed a later Ct value for RP with the multiplexed detection.

### Pooled-sample assay applicability, analytical sensitivity

**Figure 4:**
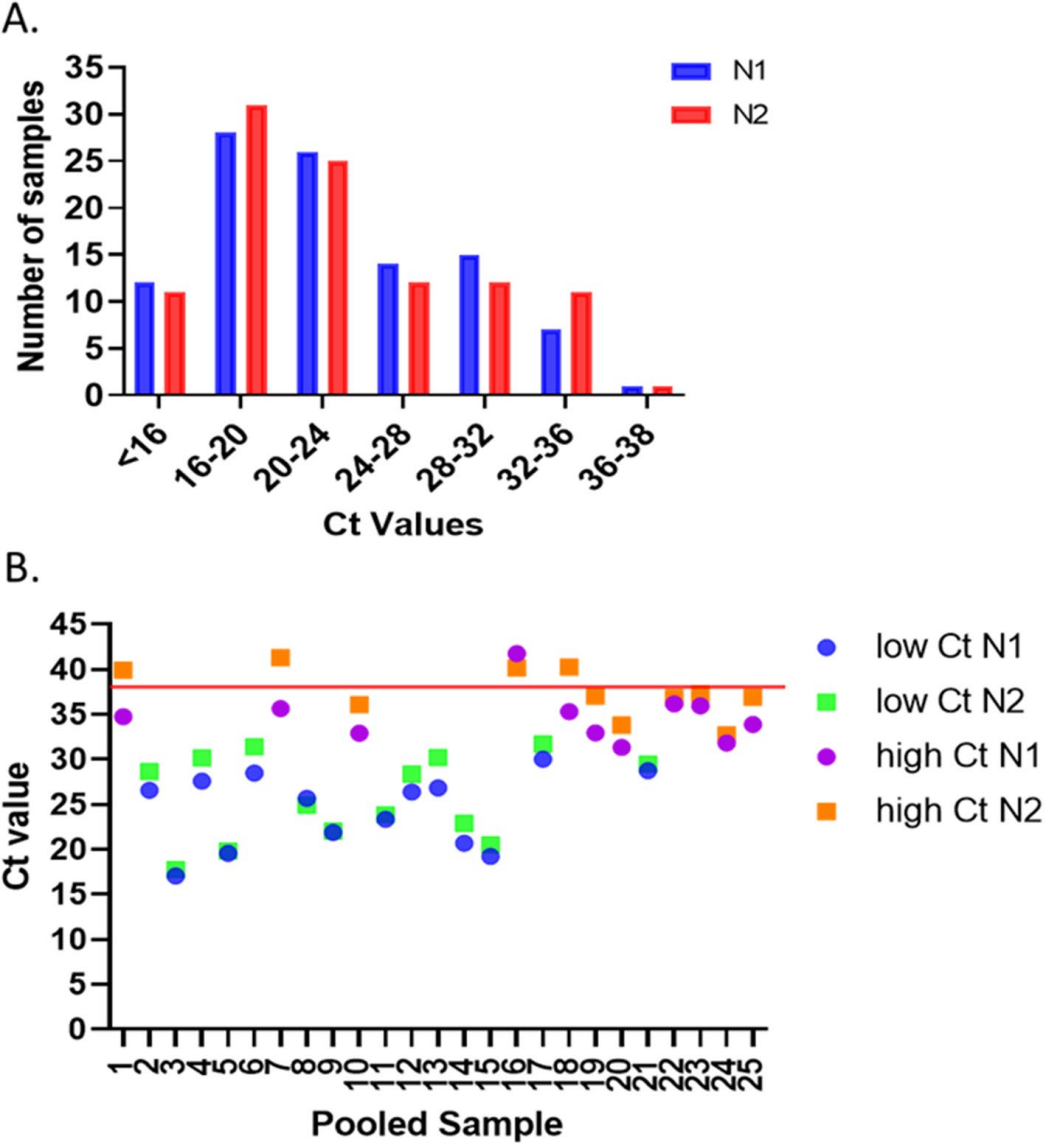
5-sample pool SARS-CoV-2 RT-PCR assay development. (**A**) Analysis of the first 103 positive samples from 2021 binned to visualize N1 and N2 Ct level. (**B**) Pools of 5 samples containing one positive sample per pool. Positive samples were chosen to represent the occupational sample population (14 low Ct samples) and to examine the effect of 5 sample pools on high Ct samples (11 samples), which could be missed due to the Ct delay caused by pooling.

A pool size of 5 samples results in a theoretical delay of 2.3 Ct. To determine the applicability of pooling 5 samples together prior to analysis to our occupational population, data from 103 positive samples collected in Jan 2–Feb 2, 2021, were examined. During this time frame, the Ct values for both N1 and N2 tended toward low Ct values (Fig. 4A), with 68% of the samples presenting with a Ct less than 25, 82.5% presenting with a Ct less than 30 and 93.2% with a Ct of less than 34. To determine what number of the 103 samples would have been missed in a 5-sample pool, 2.3 Ct was added to the sample N1 and N2 Cts and these new values were assessed as positive, negative, or inconclusive. With the addition of 2.3 Ct to simulate 5-sample pools, 101 of the positive samples would have been correctly identified as positive, 1 sample would have been identified as inconclusive and 1 sample would have been incorrectly identified as negative. As per the standard protocol, the inconclusive would have also resulted in each of the 5 samples within the pool being assessed individually, therefore this sample would most likely have been found positive on the secondary analysis. The 5-sample pooling would have resulted in correct identification of 102 of 103 samples, resulting in a positive agreement of 99.0% (95% confidence interval 94.7–99.8%).

5-sample pools were created as described in the methods section, and analysis was performed in a blinded fashion. All 25 negative pools showed no signal in N1 or N2 and were correctly identified as negative (negative agreement 100%, 95% confidence interval 85.1–100%). Of the 25 positive samples, 21 were correctly identified as positive, 3 were identified as inconclusive and 1 was identified as negative (Fig. 4B, Supplemental Table 1). All 3 inconclusive samples and the false positive sample were from weaker positives, with Ct values for N1 and N2 beyond 30 when assessed individually. The three inconclusive pooled samples all had N1 Ct values below 38 but N2 Ct values above 38. When assessed individually, 2 of the 3 positive samples in inconclusive pools had N2 Ct values above 34 (34.76 and 34.67) and the third was ~ 33 (33.08). When assessed individually, the positive sample in the false negative had N1 and N2 Ct values of 30.82 and 30.18 respectively. As the inconclusive samples would have triggered analysis of all 5 samples in each pool individually, these samples would have been most likely correctly identified on reanalysis. Therefore 24 of 25 samples would have been correctly identified and the positive agreement was 96.0% (95% confidence interval 80.5–99.3%). Finally, we compared the Ct values for N1 and N2 of the individual samples to the Ct values in the 5-sample pools (Supplemental Fig. 2). As expected, a delay in Ct was noted in the pooled samples.

## Discussion

SARS-CoV-2 is likely to be a continued presence in human populations for the coming years and has the potential for periodic outbreaks even as vaccination levels increase. In occupational settings in which work cannot be conducted remotely and/or critical processes must be maintained, rapid identification of outbreaks and infected individuals facilitates isolation of infected individuals and contact tracing to control the spread. At the start of the pandemic, the major limitation on widespread testing included insufficient reagent supply and limited testing capability^16^. Together, the limitations meant high specificity, sensitive and rapid turnaround testing for COVID infection was not readily available and available testing necessarily remained focused on testing of the symptomatic public. To aid in control of the pandemic, social distancing and stay-at-home orders were widely used to reduce contact and control the spread of COVID-19, yet these were not feasible for critical occupational settings that required in-person work^11–13^. The limited testing availability delayed time to results which could lead to large increases in cases within an occupational setting^17^. The increase in cases could be greatly minimized if the results could be obtained in ~ 48 h and quarantine and contact tracing initiated^17^.

### Singleplex assay development

Due to the need to rapidly begin occupational testing for COVID-19 but avoid resource competition with state and private laboratories, we developed a modified assay based on the CDC EUA 200001^14^. Modifications to this diagnostic panel were common and have demonstrated the sensitivity and wide applicability of the original CDC developed assay^21^. To reduce the complexity and increase the speed of assay development, the primers and probes developed by the CDC for their EUA were utilized in our assay^20^. These primers and probes were readily available throughout the pandemic and are still available now after the CDC EUA200001 has been phased out. However, the one-step RT-PCR mix and the RNA extraction reagents were experiencing significant delays in shipment (2–6 months) and therefore different reagents were chosen to avoid supply chain issues. While these changes appear minor, even minor changes can have a significant effect on assay performance and required thorough validation prior to applying to the FDA for an EUA and assaying clinical samples. Within the EUA application, the LoD was defined by the FDA as the concentration at which a minimum of 19 out of 20 samples are reliably detected, so this definition was utilized in the assay development. As 31.25 copies/reaction was successfully detected in 19 of 20 samples in two independent studies, the LoD for the singleplex assay developed was determined to be 31.25 copies/reaction (Fig. 1B). This LoD is in the same range as previously published results with the CDC developed N1 and N2 primers and probes^22,23^. For example, a previous study demonstrated detection of 20 out of 20 samples at 63 copies/reaction and 17 out of 20 samples at 31.5 copies/reaction^22^. Furthermore, a formal comparison of blinded samples conducted by the FDA across authorized EUAs, demonstrated the singleplex assay to be slightly more sensitive than the original CDC panel and comparable to many other tests^24^. Additionally the LoD was validated in contrived and clinical samples with a 100% negative sample agreement and 96.67% positive percent agreement in both studies. The percent positive agreement was calculated considering the inconclusive results as false negatives. However, in a true clinical setting, any inconclusive sample would have been retested and could have resulted in an inconclusive sample being identified as positive.

#### Increasing assay throughput

From April 2020 to June 2021 the singleplex assay was utilized for occupational testing to rapidly identify COVID positive cases, to test workers prior to or following travel, and to facilitate contact tracing to safeguard personnel. The typical workflow is described in the methods section. As the pandemic continued and more samples required analysis daily, the throughput of the singleplex assay became limiting. For a 96 well plate, only 29 clinical samples could be assessed at a time. Several additional methods were considered including dropping one of the SARS-CoV2 targets (N2), multiplexing, and sample-pooling to reduce resource use and maximize throughput. While SARS-CoV-2 detection is possible with only a single target, such as N1, two targets are recommended to provide a confirmation of SARS-CoV-2 presence in clinical sample, therefore this option was not pursued.

Multiplex assays for SARS-CoV-2 have been developed both for detection of multiple respiratory illnesses and for detection of multiple SARS-CoV-2 sequences^15,25–30^. While differentiating SARS-CoV-2 from other respiratory illnesses that present with similar symptoms is highly important in healthcare settings^15,28–30^, the major goal of the occupational testing was to identify SARS-CoV-2 positive individuals in order for contact tracing to be initiated, therefore we focused on multiplexing SARS-CoV-2 detection alone. A simple methodology to maximize throughput was the development a multiplex assay format that would allow detection of all 3 targets in a single well using spectrally distinct fluorophores. This would allow 93 samples to run in a single 96-well PCR plate but does require RT-PCR instrumentation capable of detecting 3 or more colors. To expedite this development and FDA authorization, changes to the existing singleplex assay and supply chain needed to be minimized; therefore only the fluorophore on the N2 and RP probes was changed. To qualify as equivalent to the original EUA^20^, the new LoD must be within threefold of the originally determined LoD. Initial results using equal amounts of all three probes did not achieve this criterion. The Ct delay for N2 was so significant that positive detection was not reliable even at 4 × LoD. Two potential options were considered, the first was to switch genes for the second SARS-CoV-2 target and the second was to attempt to optimize the current target set. As altering the gene target would require a completely new application to the FDA rather than a modification of the current EUA, which would delay the use of the multiplex assay, an attempt was made to optimize the current target set. The N1 and N2 targets are within the same gene and present at the same level in the sample. In an ideal reaction these two signals should be equivalent, however, the N2 primer/probe has proven less than ideal at the standard cycling conditions used in the assay^31^. While this can be mitigated by adjustment in the PCR parameters^31^, specific parameters for each primer and probe set are not compatible with multiplexing so we tested a modification that was compatible with multiplexing.

Interestingly, no delay was noted in the positive control, which has the equivalent amount of SARS-CoV-2 synthetic RNA as the 4 × LoD but much lower levels of RP than clinical samples. This issue, in which a target expressed at high levels results in poor performance of the lower targets, is a common problem for multiplexed PCR^32^. As the high abundance target in this PCR is the endogenous control (RP), we tested if limiting the RP primers and probes would result in increased performance. A reduction of RP primer and probe concentrations at 1/3 the concentration of N1 and N2 resulted in a slight delay in the Ct for RP, but 100% percent positivity agreement in both technical replicates of 20, confirming that multiplexing the assay was possible without altering the LoD. This LoD of 31.25 copies/reaction is similar to or better than previously reported LoDs for multiplexed SARS-CoV-2 detection^26,27^. In all cases the slightly delayed RP was still well below the cutoff for a valid sample and is not expected to affect the clinical result given the criteria for interpretation of clinical results presented in Table 1.

Beyond multiplexing, sample pooling presents an alternative way to increase sample throughput and has the potential to allow even more samples to be processed than multiplexing. With 3 targets, the maximum sample capacity per plate of the multiplexed assay is 93. In comparison, any pool size over 3 would result in increased sample throughput compared to multiplexing. Multiple pooling strategies have been explored for SARS-CoV-2 testing. In the simplest strategies, often referred to as a 2-stage strategy, a number of samples (typically 3 or greater) are pooled and assessed together^33–36^. If the pool is negative, all samples within the pool are considered negative and no further testing on these samples is needed. If a pool is positive, a second stage of testing is performed, where all the individual samples are then tested to determine which sample or samples are positive. The 2-stage strategy can have significant improvement in resource utilization, however the improvement in resource utilization is tied to the positivity rate, as a second round of testing is required for all samples in a positive pool^36–39^. Typically a 2-stage pooling strategy is appropriate in expected positivity rates of 10–15% depending on pool size. In the case of extremely low expected positive rate, such as 1–5%, further resource improvement can be attained by a multistage approach in which three stages are used to facilitate testing large pools of samples at once. For example, starting with a pool size of 16 samples for stage 1 and then retesting positive pools in a pool of 4 for stage 2 and going to single sample testing only at stage 3 resulted in a theoretical improvement of 3 to 7 times the numbers of individuals tested with prevalence rates of 5 and 1% respectively^37^. While a 3-stage approach can result in significant resource savings, the 3 stages of testing would result in a delay in time-to-result from collection that was longer than 24 h targeted for our occupational setting. Other methodologies, such as matrix pooling are also highly effective but are commonly done with programmed robotic liquid handling not readily available for occupational testing^33,40^. Therefore, we chose to focus on traditional 2-stage pooling.

While pooled sample analysis facilitates higher sample throughput, pooling samples can reduce the sensitivity of the assay by diluting the positive target and this effect is related to the number of pooled samples. For example, pooling of 32 samples resulted in an estimated false negative rate of 10%^41^. The rate of detection of pooled positives is also related to the strength of the positive signal. A study of 10 sample pools found that 29 of 253 ‘negative’ pools were false negative, a rate of 11.5%^34^. Upon closer examination a missed positive was 3 times more likely to occur with a high Ct positive (Ct > 35) than with a low Ct positive sample^34^. The increased rate of false negatives with weak positive samples (high Ct) is because these samples are already close to the positive cut off, and there is an expected theoretical Ct delay of Log_2_(X), with X being the number of pooled samples. This theoretical delay can be calculated based on the number of samples pooled and compared to the current Ct distribution of the population assessed on an individual basis. The other information needed to examine the population is the pool size; the prevalence rate of SARS-CoV-2 in the population has a strong impact on the efficiency of pooled sample analysis. The prevalence in our occupational population ranges from 1 to 12% (Fig. 2), which when compared to pool predictions suggests an optimal pool size of 10 for 1% and 3 for 12% and a pool size of 5 for the range of 4–6% prevalence^42,43^. As the pool size of 10 had the potential for a false negative rate above our acceptable range and a pool size of 3 provides only minimal advantage over the multiplex assay, we focused on a pool size of 5, which would cover the median prevalence rate found in our population. Finally, we examined the possibility of pooling samples before extraction^34,35,41^ or after RNA extraction^42,44,45^. Pooling prior to extraction provided additional resource savings in the extraction step, therefore we chose to pool samples prior to RNA extraction.

Following the determination that 5-sample pooling would be appropriate for the current occupational population, pooled samples were tested. The samples were selected to represent three potential populations to increase our understanding of how the test would perform in various situations following the current guidance from the FDA for developing a pooled sample assay. The first population was negative samples, which represents the largest portion of the samples received in the occupational laboratory. The second population was designed to assess most positive samples present in our population, with Ct below 30. The third population was designed to represent low positive samples, with Cts above 30. In examining the data from the positive pools, the 14 of 14 low Ct samples which represent 82.5% of the occupational population were correctly identified (Fig. 4B) and is consistent with other studies of detection of strong positive samples with pooled samples^33,34,46^. However, similar to other studies^33,34,47^, as the Ct values become higher, the rate of inconclusive and false negative increases. Taken together, this data suggests that Ct value in the positive population should be monitored over time to reduce the likelihood of missing a positive sample.

Similar to previous studies^41,42,47^, the SARS-CoV-2 target Cts were delayed in the pooled samples. N1 showed an average Ct delay of 1.5, whereas the N2 was delayed by an average of 2.6 Ct. This more significant impact on N2 with pooling was also visible in the Ct of the 5-pool samples as all the inconclusive samples were N1 positive but N2 negative (Fig. 4B). The level of Ct delay is similar to previous studies which found a delay of 2.2 in N2 and 2.7 in N1 with 5 sample pools^41,42^.

#### Factors to consider when deciding on the use of multiplexing and pooling in occupational testing

In occupational testing, there are several potential testing populations with potentially different testing requirements. There are 3 high risk populations, namely those who are symptomatic, those who have had known contact with a positive individual or those who have traveled. There is also the potential need for screening of individuals with no known risk factors^48,49^. Decisions on which population(s) to test and how frequently can have significant impact on the needed throughput. For example, modeling suggests that for it to be effective, routine screening may need to occur between every 2 days to weekly^17,50–52^.

Table 2 provides an assessment of the sample throughput and time to perform the assay for the three approaches we present in this paper.

**Table 2:** Overview of analysis methods.

Method	Number of patient samples/96-well plate	Time per run, manual extraction	Time per run, automated extraction
3 target singleplex PCR	29	4–5 h*	3 h
3 target multiplex PCR	93	6–7 h*	3 h
5-sample pool combined with 3 target singleplex PCR	145	4–5 h*	3–4 h#

*Manual extraction allows extraction of 23 samples at a time at ~ 1 h per round. # Automated extraction for samples combined prior to RNA extraction which allows for extraction of 96 samples at a time.

**Figure 5:**
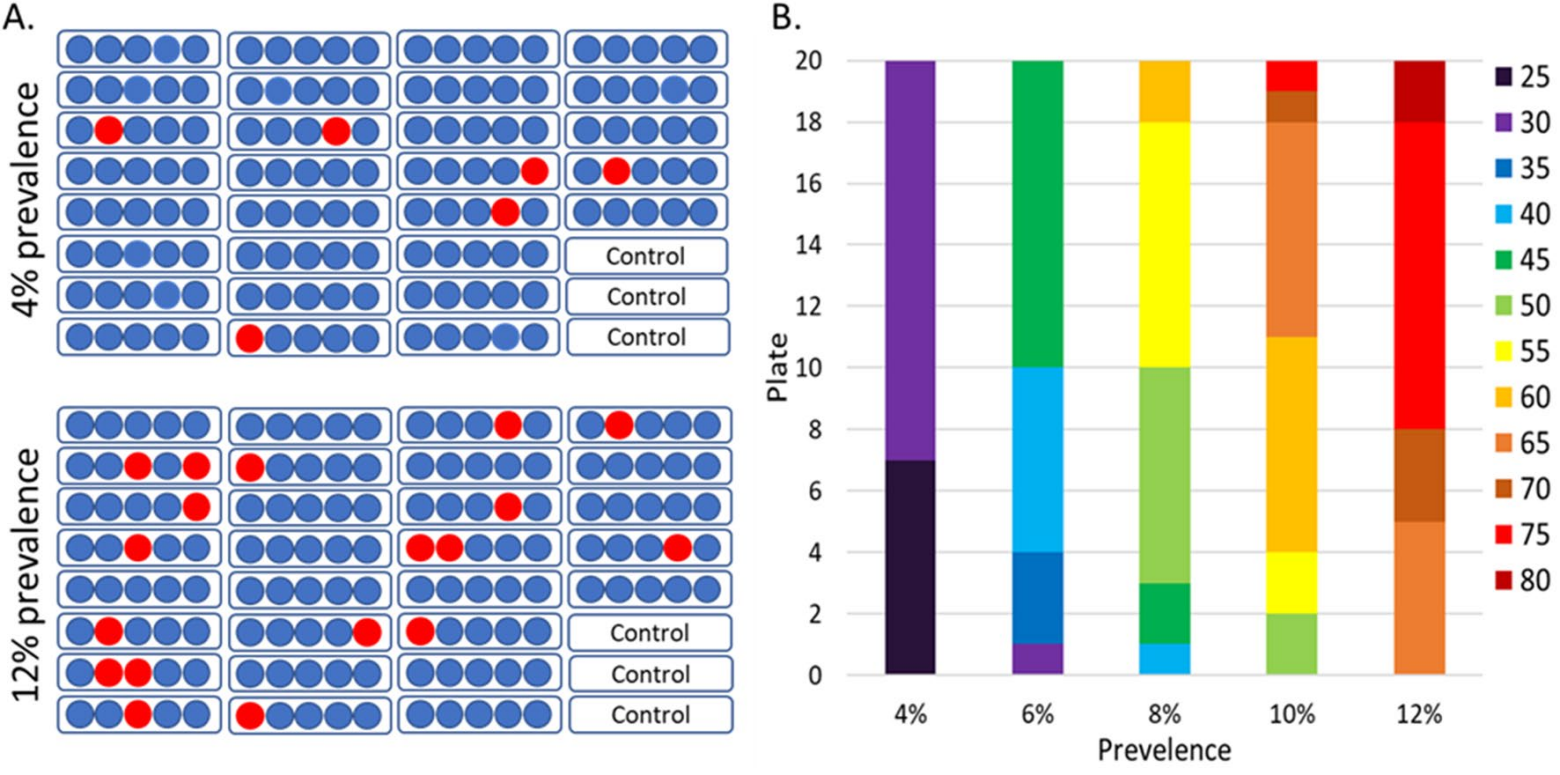
Effect of prevalence rate on the number of samples that require repeat testing. (**A**) Visual display of the first predicted plates for 4% and 12% prevalence in the testing population. The 12% prevalence illustrates the increased likelihood of multiple positives being pooled randomly into the same well. (**B**) 20 plates were predicted for each prevalence level using random number generation to examine the effect of prevalence on the number of samples that required reanalysis. The colors indicate the number of samples that require rerun of samples independently to identify the positive sample based on 5-sample pools.

For a 3 target RT-PCR, 5 sample pooling is more efficient than multiplexing as pooling allows 145 samples to be run simultaneously while still maintaining the appropriate controls on each plate. Additionally, pooled samples, when pooled prior to extraction save on time, labor, and reagents by reducing the number of extractions, as all 145 samples are covered by 29 extractions. However, the efficiency of pooling is highly affected by the positivity rate. Examining prevalence rates seen in our population (Fig. 2) and how many plates would be required to address 145 samples, we predicted the relationship between positivity rate and retesting frequency. To reflect the random pooling of positive and negative samples, a random number generator was used to select the positive samples. In the case of a low positivity rate of 1%, only 1–2 samples would be positive out of the 145, resulting in a maximum set of 10 samples for retesting. As the positivity rate increases to 4%, 5–6 samples should be positive (Fig. 5A,B). Using 6 samples per 145 as a basis, the prediction of 20 random plates found 35% of the plates would have 5 positive wells and 65% of the plates would have 6 positive wells requiring 25 to 30 samples to be retested. As the prevalence of SARS-CoV-2 increases within the tested population, the likelihood of multiple positive samples being randomly assigned to the same pool increases (Fig. 5B). At 12% prevalence, so many wells overlapped that none of the plates generated had all single positive wells and most of the plates resulted in 15 positive wells, necessitating retesting 75 of the 145 initial samples. Even with a 12% prevalence and retesting of approximately half the samples, by pooling prior to extraction, resources are still saved in the extraction using the pooling methodology. Pooling has an added advantage in that it can be run on most real-time PCR machines unlike the multiplex assay.

#### Optimal, hybrid approach to facilitate both high-priority testing and routine screening

**Figure 6:**
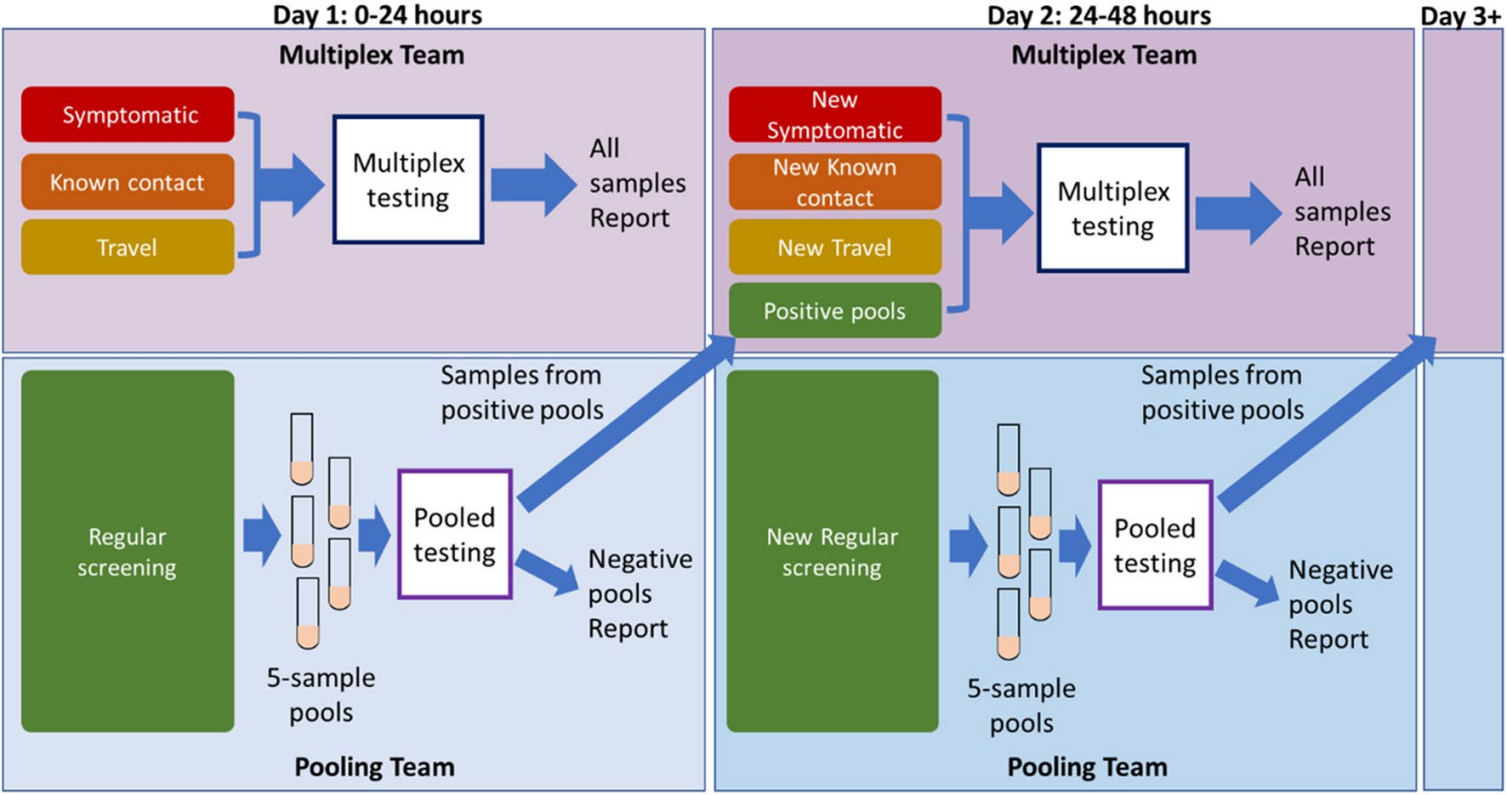
Proposed efficient hybrid testing design using two teams to perform both multiplex testing and pooled testing simultaneously. Day 1 denotes the first day of any testing run, for example a Monday or a week. To maintain ~ 24 time to results, all higher risk samples such as symptomatic, known prior contact with a positive individual and recent travel would be run using the multiplex testing. Regular screening would be run by a second team using the 5-sample pooling method. Negative samples from the screening population would be reported the same day. Samples from the positive pools would be set aside and run with the Day 2 multiplex samples. In this way, all samples would report out in ~ 48 h and all high-risk samples would report out in ~ 24 h from collection.

While pooling saves on resources and expands the instruments that can be used, the potential to rerun many samples can result in a significant delay in results. Multiplex testing can address the time to result effectively, but is more resource intensive, less efficient (Table 2), and requires specialized multicolor instrumentation not common in all laboratories. Given the relative advantages of each methodology and the need to test 3–4 potential populations, we designed a hybrid approach to testing with teams running both multiplex and pooled testing simultaneously (Fig. 6). The first three populations are the highest risk groups, specifically individuals who are symptomatic, have known contact with a COVID positive individual, or have returned from travel. As this population is the most likely to have positive individuals, it is also the most time-sensitive to facilitate initiation of quarantine and contact tracing to prevent spread within the workplace. Therefore, we propose to use multiplex testing to examine these individuals, as the sensitivity is higher and the time to result is lower than pooled samples. These three populations represent the standard population tested at our institution at the peak of the pandemic. Examination of the sample inflow typical at the highest need (Fig. 2) would only require 90–150 samples a day in our occupational setting. Using the multiplex method, this level of analysis would require only 1–2 PCR plates a day.

The addition of pooling to the testing scheme allows the inclusion of a screening population due to the reduction in resource utilization and personnel needed for extraction. Large-scale pooling has demonstrated a savings of 76% of the RNA extraction and RT-PCR resources even with a variable prevalence rate of 0.5–6%^36^. The inclusion of a screening population could be vital as modeling has suggested screening is much more effective than symptomatic testing for controlling the spread of SARS-CoV-2 infection^17,52^. It is important to note that the efficiency of pooling is highly dependent on the current positivity rates as a single positive would require the whole pool to be retested. By separating out the routine, low risk screening population from the higher risk group, we hope to maximize the resource benefit and limit the number of pools the require reanalysis (Fig. 6). A very large systematic review and meta-analysis of SARS-CoV-2 testing of asymptomatic people found the positivity rate to be only 0.25% in the general population and increasing only to 2% among travelers^53^, suggesting that screening would result in only 1 or 2 positive wells necessitating only 5–10 samples be retested. These samples could easily be worked into the multiplex workflow on the second day (Fig. 6). At this rate, 98–99% of the screened personnel and 100% of the high-risk personnel would receive results in ~ 24 h. By splitting the populations across two testing methods and personnel teams, we can meet the rapid test turnaround time and the potential screening needs to help protect a in person workforce. The hybrid scheme is flexible to address the changing needs of SARS-CoV-2 testing. For example, in a highly vaccinated population where positivity prevalence is expected to be low, the sample pooling fraction could be increased. As seasonal changes result in increases or decreases in viral prevalence or a new variant emerges, a shift to the more sensitive multiplex assay would increase efficiency. In the designed workflow either multiplex testing or pooling could be increased, reduced, or completely halted without affecting the other team.

## Conclusion

To address the needs within an occupational population that must maintain an on-site work presence during the SAR-CoV-2 pandemic, we set up our own diagnostic laboratory in existing biological research laboratories and developed our own test based on the available CDC 2019-nCoV Real-time RT-PCR Diagnostic Panel (EUA200001) but designed to minimize competition for reagents with current testing of the general population^14,20^. Throughout the early phases of the pandemic we successfully tested symptomatic, known contact, and travelers within our occupational population with a ~ 24–48 h turnaround time to facilitate quarantine, contact tracing, and limit the spread of COVID-19. As the pandemic progressed and the testing load increased, we developed modifications of our assay to include 5-sample pools and multiplex testing. We showed these methods could maintain necessary sensitivity while increasing the throughput. Finally, we present a hybrid testing strategy using both 5-sample pooling and multiplex testing to address the unique needs of an occupational setting for both rapid analysis of personnel at high risk of COVID infection and routine screening. This hybrid testing strategy provides flexibility to shift between the multiplex and pooling strategies to adjust for changes in expected positivity rates as the pandemic progresses. Although the scope of this work was on nasopharyngeal swabs, the SNL-NM 2019 nCoV Real-Time RT PCR Diagnostic Assay (EUA200481) is authorized for use with other upper respiratory samples. Additionally, the multiplex, pooling, and testing strategies could be extended to other PCR-based diagnostic assays for upper respiratory samples or even saliva-based assays with proper validation.

The work presented here has potential impact beyond the current pandemic. Occupational testing has previously been recommended in healthcare workers for other illnesses including Tuberculosis and Hepatitis B^54–56^. However, these tests are typically run a maximum of once to twice a year per worker. The current SARS-CoV-2 pandemic has presented occupational workers with a first-ever requirement for regular and on-going testing. For example, the Centers for Medicare and Medicaid Services recommends routine testing for staff who are not up to date with all recommended COVID-19 vaccine doses in Long-Term Care Facilities between twice a week and once a month, depending on the prevalence of COVID-19 in the local population^57^. The frequency of testing required for a fast spreading respiratory virus combined with the complexity of the testing and the issues presented by the pandemic has presented obstacles to safety of occupational populations. The approaches presented in this paper, such as how to effectively bring a laboratory test online to avoid supply chain restrictions and how to successfully modify tests for increased throughput, provide a blueprint for future responses.

## Supplementary Information

Supplementary Information.

## Data Availability

The data underlying this article will be shared on reasonable request to the corresponding author.
