# Supplementary material for: Singleplex, multiplex and pooled sample real-time RT-PCR assays for detection of SARS-CoV-2 in an occupational medicine setting
Source: Sci Rep. 2022 Oct 22;12:17733. doi: 10.1038/s41598-022-22106-2 (PMC9587995; doi:10.1038/s41598-022-22106-2)
Supplement: Supplementary file 1 — Supplementary Information. [file 41598_2022_22106_MOESM1_ESM.docx]

“Singleplex, multiplex and pooled sample real-time RT-PCR assays for detection of SARS-CoV-2 in an occupational medicine setting”

Kimberly S. Butler^1^, Bryan D. Carson^1^, Joshua D. Podlevsky^1^, Cathryn M. Mayes^2^, Jessica M. Rowland^3^, DeAnna Campbell^4^, J. Bryce Ricken^1^, George Wudiri^5^, The SNL COVID Dx Assay Team, Jerilyn A. Timlin^1,6^*

*Corresponding author [jatimli@sandia.gov](mailto:jatimli@sandia.gov)

^1^Molecular and Microbiology Department, Sandia National Laboratories, Albuquerque, NM 87123 United States

^2^WMD Threats & Aerosol Science, Sandia National Laboratories, Albuquerque, NM 87123 United States

^3^Global Chemical and Biological Security, Sandia National Laboratories, Albuquerque, NM 87123 United States

^4^Biological and Chemical Sensors Department, Sandia National Laboratories, Albuquerque, NM 87123 United States

^5^Cooperative Nuclear Counterproliferation, Sandia National Laboratories, Albuquerque, NM 87123 United States

^6^Computational Biology and Biophysics Department, Sandia National Laboratories, Albuquerque, NM 87123 United States

**SUPPLEMENTAL TABLE**

**Supplemental Table 1: Ct values inconclusive and false negative pooled samples**

| **Pooled sample** | **Pooled sample N1 Ct** | **Pooled sample N2 Ct** | **Positive sample un-pooled N1 Ct** | **Positive sample un-pooled N2 Ct** |
| --- | --- | --- | --- | --- |
| Inconclusive 1 | 34.74 | 39.9 | 32.15 | 34.76 |
| Inconclusive 2 | 35.63 | 41.29 | 32.71 | 34.67 |
| Inconclusive 3 | 35.31 | 40.27 | 33.61 | 33.08 |
| False Negative | 41.76 | 40.15 | 30.82 | 30.18 |

**SUPPLEMENTAL FIGURES**

**
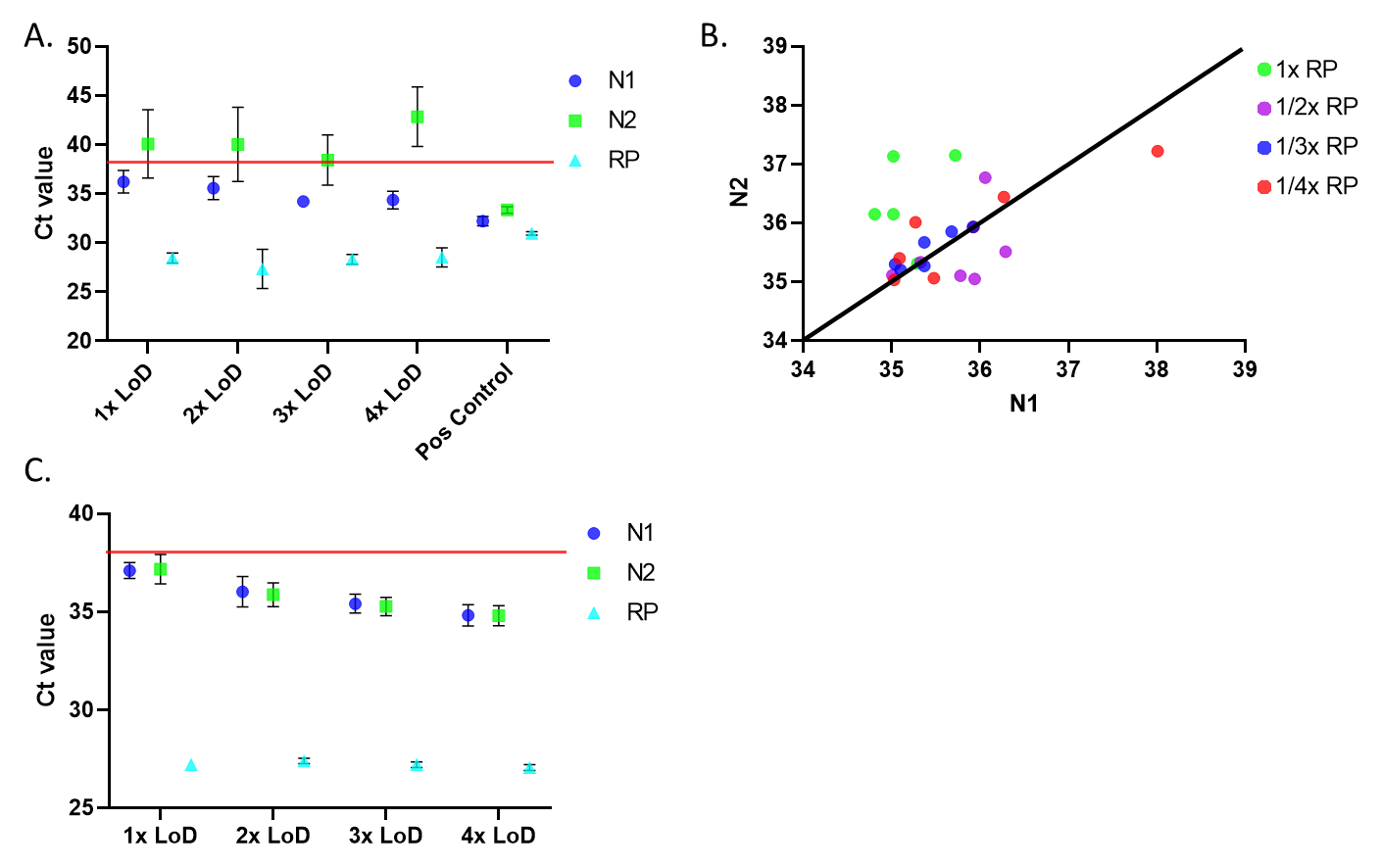
**

**Supplemental Figure 1. SARS-CoV-2 RT-PCR multiplex assay development.** Assay detects 2 sequences (N1 and N2) from SARS-CoV-2 and a human RNA (RP) as an internal extraction control. A) Initial test of multiplex detection near the LoD of SARS-CoV-2 synthetic RNA spiked into pooled negative nasopharyngeal samples at known copy numbers. Positive control contains all three targets at similar levels and the SARS-CoV-2 synthetic RNA at the same level as the 4x LoD. Primer and Probe concentrations were the same as the singleplex assay (Primers at 500nM and probes at 125 nM). N=12, error bars represent the standard deviation. Line denotes the cut off for positivity (38 Ct). B) Test of reduced RP primers and probe on N1 and N2 Ct. Samples consisted of synthetic SARS-CoV-2 RNA spiked at 2x LoD in pooled negative nasopharyngeal samples. N=6. Line represents equivalent signal from N1 and N2. C) Test of multiplex detection near the LoD of SARS-CoV-2 synthetic RNA spiked into pooled negative nasopharyngeal samples at known copy numbers. RP primers and probes were utilized at 1/3 the level of the single plex assay. N=12, error bars represent the standard deviation. Line denotes the cut off for positivity (38 Ct).

**
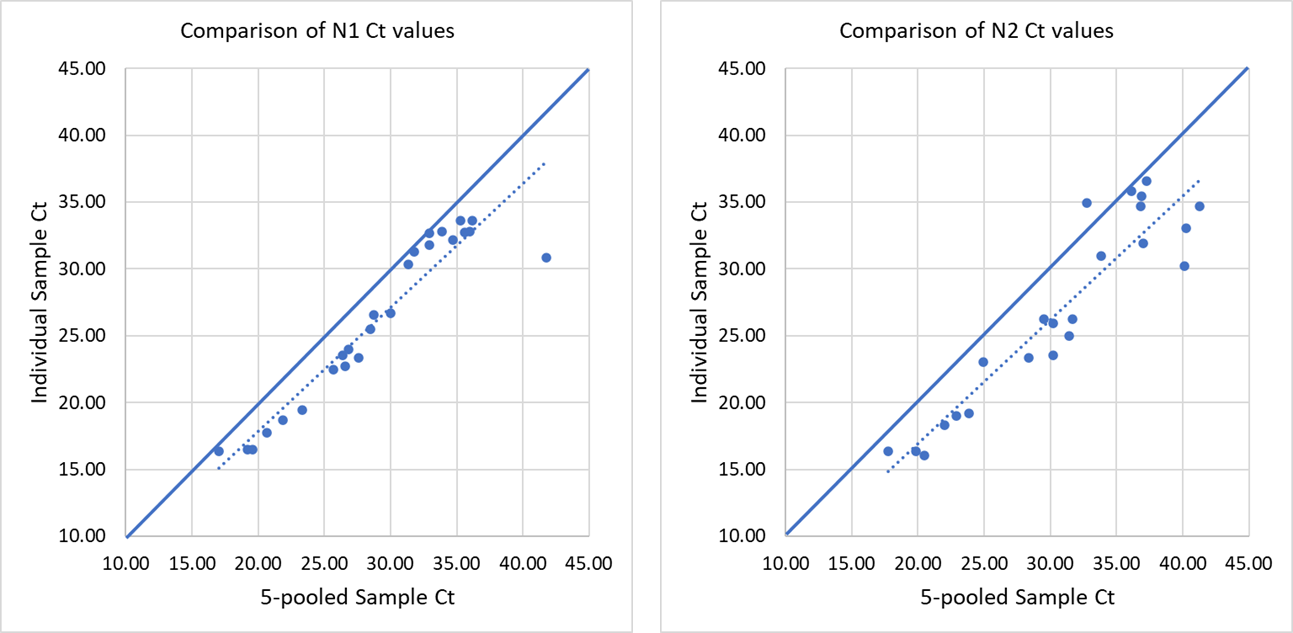
**

**Supplemental Figure 2. Comparison of individual positive sample results to the result of the same sample in the 5-sample pool for N1 and N2.** Each 5-sample pool was composed of a single positive with 4 negative samples. The solid line represents a 1:1 relationship which would denote no Ct delay. The dotted line is the linear regression of the data.
